# Special Issue “Epigenetic Genes, Biomarkers and Immunotherapy in Cancers”

**DOI:** 10.3390/ijms27177628

**Published:** 2026-08-26

**Authors:** Urszula Oleksiewicz

**Affiliations:** Department of Cancer Immunology, Poznan University of Medical Sciences, Rokietnicka Street 8, 60-806 Poznan, Poland; oleksiewiczu@ump.edu.pl

Malignant transformation is largely driven by the accumulation of genetic mutations that confer proliferative advantages, evade various cell death mechanisms, and sustain cell viability under adverse conditions. Nevertheless, it is also widely acknowledged that other internal and external cues contribute to cancer transformation and progression [1]. Internal signaling often depends on epigenetic mechanisms, which may be transient or persist over the long term through diverse inheritance mechanisms [2,3,4]. It is now widely recognized that epigenomic signatures are profoundly disrupted in many cancers [5]. Epigenetic instability may stem from genetic mutations, aberrant expression of proteins involved in epigenetic regulation, or metabolic dysfunctions [6,7,8]. Such modifications support the transcriptional networks underlying the acquisition and maintenance of cancer-associated traits. Unlike largely irreversible genetic mutations, epigenetic alterations are reversible, enabling rapid cellular reprogramming and adaptation under unfavorable conditions [3,4,9]. Epigenetics encompasses interconnected molecular mechanisms that regulate chromatin structure and function. These include DNA methylation, histone modifications, histone variants, nucleosome positioning, three-dimensional chromatin organization, and non-coding RNAs. Acting together, these processes alter DNA accessibility without changing the underlying nucleotide sequence, thereby regulating gene expression and affecting various cancer hallmarks, such as uncontrolled proliferation, resistance to apoptosis, metastatic potential, replicative immortality, and immune signaling [4,5,9].

Immune signaling is widely recognized as a key external factor that may modulate cancer pathogenesis and responses to various therapies. Although the immune system can recognize and eliminate tumor cells in a process known as immunosurveillance, cancer cells may acquire multiple mechanisms of immune evasion during tumor evolution [10]. These include loss of antigenicity through epigenetic silencing of tumor-associated and tumor-specific antigens, defects in MHC I-mediated antigen presentation, and activation of immune checkpoint pathways such as PD-1/PD-L1 and CTLA-4 [11,12,13]. In addition, the tumor microenvironment promotes immunosuppression by limiting immune cell infiltration and recruiting immunosuppressive cell populations, including myeloid-derived suppressor cells (MDSCs), tumor-associated macrophages (TAMs), neutrophils (TANs), and regulatory T cells (Tregs) [14]. As these mechanisms enable tumor cells to evade immune control and promote tumor progression, immunotherapy aimed at restoring or enhancing the immune system’s natural ability to eradicate cancer cells has become a widely used and effective therapeutic approach in recent years. Despite their promise, therapeutic responses can be compromised by tumor-associated immune suppression and adverse autoimmune reactions [10,14]. To improve the overall efficacy of immunotherapeutic approaches, substantial effort has been devoted to identifying biomarker signatures and novel therapeutic options. Many studies reveal the underlying interplay between epigenetic traits and immune signaling, highlighting the importance of both factors as tools that could be incorporated into modern strategies for cancer patient management.

The articles collected in this Special Issue, “Epigenetic Genes, Biomarkers and Immunotherapy in Cancers”, highlight the increasingly recognized interplay among epigenetic regulation, cancer cell biology, and immune system signaling. Some of the contributions focus on immune checkpoint pathways in solid tumors, emphasizing the importance of the Programmed Cell Death 1 (PD-1)/Programmed Cell Death Ligand 1 (PD-L1) axis in immune evasion and the therapeutic exploitation of this axis through immune checkpoint inhibitors [15,16]. Complementing these studies, other papers explore the epigenetic landscape of cancer, from the diagnostic and prognostic value of hTERT promoter methylation patterns [17] to the contribution of non-coding circular RNAs [18], and histone deacetylases (HDACs) in tumor progression [19]. The reported associations between the expression pattern of HDAC family members, cancer stemness, and immune cell infiltration pinpoint the notion that epigenetic dysregulation not only drives malignant transformation but also affects the composition and function of the tumor microenvironment [19]. Collectively, these studies illustrate how epigenetic and immune signatures may serve as novel biomarkers and therapeutic targets, thereby advancing precision oncology approaches to improve immunotherapeutic strategies.

More specifically, the review by Emanuela Germana and colleagues [15] demonstrates the value of PD-L1 expression assessment in guiding clinical decisions and predicting response to immunotherapy with ICIs (immune checkpoint inhibitors) in advanced bladder cancer. The article presents current classification, treatment modalities, and prognosis for three types of bladder cancer: non-muscle invasive bladder cancer, muscle-invasive bladder cancer, and metastatic tumors. As multiple studies have indicated that high PD-L1 expression frequently appears in high-grade bladder tumors, ICIs have emerged as a potential therapeutic regimen for patients with progressive disease [15]. Importantly, the authors emphasized substantial histologic, molecular, and immune heterogeneity of bladder cancer, which may impair precise diagnosis and compromise concordance between transcriptomic and immunohistochemical (IHC) evaluations [20]. Thus, several immunohistochemical assays, scoring systems, and cut-off points have been established to assess PD-L1 expression prior to ICI administration [21,22,23]. These assays were used in several clinical trials that have been comprehensively presented in the review. The trials tested various ICIs in the cohorts with advanced urothelial bladder cancer [15]. Interestingly, a few of the reported studies showed significantly improved ICI efficacy in patients with high PD-L1 expression [24,25]. However, some trials reported benefits of ICI regardless of PD-L1 status, while others showed limited efficacy of ICI compared with standard treatment in metastatic tumors [26]. Although the overall data indicate the effectiveness and safety of ICI treatment in progressive bladder tumors, the authors emphasized that tumor heterogeneity and changes in the dynamic TME composition and cellular epigenomics may account for the limited predictive power of current PD-L1 assays [15].

Immunotherapeutic approaches were also described in the context of gastric cancer [16]. In their narrative review, the authors synthesized current information on the epidemiology and etiology of gastric cancer, highlighting preventive measures, clinically relevant biomarkers, and available treatment options. Various forms of ICI immunotherapy are a major focus of the review. The authors elegantly explained the molecular function of immune checkpoints, including the PD-1/PD-L1 axis and the binding of cytotoxic T-lymphocyte antigen 4 (CTLA-4) to CD80/CD86 molecules. In addition to those two well-studied signaling networks, other immune checkpoint molecules and their roles in the immune network are also listed. Cancer cells hijack mechanisms present in non-cancerous settings to evade immunosurveillance. Under non-malignant conditions, immune checkpoints reduce excessive immune responses, thereby preventing tissue destruction and autoimmunity. In cancer, however, immune checkpoint signaling impairs T cell proliferation, differentiation potential, and activity, which subsequently leads to T cell exhaustion, increased immune tolerance, and, ultimately, cancer cell evasion of immune clearance [27,28,29]. Blocking immune checkpoint molecules is therefore an attractive option to relieve immunosuppression and arm patients’ immune systems against cancerous cells [29,30]. Indeed, some clinical trials demonstrated favorable therapeutic effects in gastric cancer patients treated with various forms of ICIs either as monotherapy or in combination with other drugs, e.g., trastuzumab, an anti-HER2 monoclonal antibody [31,32,33]. Interestingly, patients meeting higher PD-L1 expression cut-offs demonstrated better response to combination therapy with ICIs [31]. Nevertheless, the article also underscores the limited ICI efficacy in some of the gastric cancer patients, e.g., in patients with liver metastasis, pinpointing the need for more precise identification of the factors responsible for the resistance to therapy [16].

Indeed, as highlighted in the perspective by Simeon Santourlidis and colleagues [17], the search for robust biomarkers is essential for the development of reliable diagnostic methods and evidence-based guidance for anti-cancer therapies. The authors focused on the utility of the epigenetic status of Telomerase Reverse Transcriptase (hTERT) as a pan-cancer biomarker to improve diagnosis, prognosis, residual disease detection, and decision-making in the context of chemotherapy [17]. The latter is particularly important for achieving optimal exposure to chemotherapeutic agents while avoiding overtreatment, thereby balancing successful eradication of cancer cells with reduced cytotoxicity to normal tissues [17,34]. Altered methylation profile in the long hTERT promoter region may be successfully assessed using cancer-derived DNA, including cell-free DNA (cfDNA) released into the tumor-surrounding body fluids from dying cancer cells [17,35]. Notably, a liquid biopsy may be collected in a minimally invasive manner. Interestingly, hTERT is broadly expressed across multiple tumor types, as observed in 73% of 6835 tumors [36], thereby enabling the acquisition of replicative immortality [1]. Multiple studies indicate that hTERT re-expression is, at least in part, driven by epigenetic mechanisms [36]. In many tumors, the proximal hTERT promoter region is hypomethylated, whereas the distal region is, counterintuitively, hypermethylated [37,38]. The hypermethylated locus, dubbed TERT Hypermethylated Oncological Region (THOR), encompasses 52 CpG sites upstream of the TERT core promoter [37]. The data suggest that THOR contains sequences that regulate the expression of an antisense promoter-associated (hTAPAS) lncRNA, which may be responsible for the post-transcriptional silencing of hTERT expression [39]. Thus, it has been proposed that hypermethylation within the hTERT distal THOR locus represses hTAPAS, which in turn leads to the hTERT re-expression and, ultimately, to cancer cell immortalization [39,40]. Given the pan-cancer phenomenon of activated hTERT expression, the authors concluded that assays measuring the methylation profile of the hTERT promoter, e.g., via methylation-specific PCR, may be useful in various clinical contexts [17].

While DNA methylation is a classical epigenetic marker, Karolina Włodarczyk and colleagues, in their review [18], highlighted cancer-related aspects of circular RNAs (circRNAs), an emerging and rapidly evolving field of epigenetic research. The authors focused mainly on the impact of circRNAs in endometrial cancer (EC). After a detailed overview of EC epidemiology, risk factors, genetic background, heterogeneity, staging, and molecular classification, the authors explored multiple aspects of circRNA research. CircRNAs, formed from pre-mRNA via back-splicing, may contain a variable composition of exonic, intronic, and/or intergenic sequences. They lack a 5′ end cap and a 3′ end poly(A) tail, and both ends are covalently connected, generating a circular entity [41]. Such a structure protects against RNase R exonuclease digestion, thereby rendering circRNAs highly stable [42]. They may arise via different biogenesis mechanisms, including exon skipping, intron pairing, interaction with RNA-binding proteins, tRNA-dependent circularization, and other mechanisms [41]. Some circRNAs act as miRNA sponges, sequestering miRNAs and preventing their interaction with target mRNAs, thereby promoting mRNA stability and translation. For instance, in EC, hsa_circ_0002577 binds miR-197, which is responsible for CTNND1 silencing, thereby mediating Wnt/β-catenin signaling and, consequently, cell proliferation, migration, and invasion [43]. CircRNAs may also sponge RNA-binding proteins, as shown with circCHD7. CircCDH7 binds insulin-like growth factor RNA-binding protein 2 (IGFBP2), thereby activating JAK/STAT signaling and promoting proliferation in EC cells [44]. Furthermore, some circRNAs regulate parental gene transcription through interaction with RNA polymerase II (e.g., EIciRNA in the complex with U1 snRNP) [45]. The review systematically compiled current evidence on various circRNAs dysregulated in EC, along with their reported molecular and functional properties. Application of circRNAs was also discussed in the context of immunotherapy [18]. For example, circRNAs may serve as carriers of antigen-encoding sequences, similarly to mRNA-based cancer vaccines, but with superior stability. They may also be used to modulate the expression of immune checkpoint proteins or other immune modulators to improve ICI- or CAR-based therapies [46,47]. Despite growing interest in circRNAs, further research is needed to develop robust circRNA-based methodologies for clinical application.

As mentioned earlier, epigenetic dysregulation is a crucial component modulating tumor biology. This phenomenon was also confirmed in the study on the histone deacetylase (HDAC) signature in solid tumors [19]. HDACs enzymatically remove acetyl groups from histone and non-histone proteins [5,48]. From the epigenetic perspective, histone deacetylation promotes chromatin compaction due to increased affinity between DNA and histones, thereby impairing transcriptional activity within an affected region [5,48]. Based on their homology, structure, and cofactor usage, HDACs are divided into four classes: class I (comprising HDAC1/2/3/8), class II (HDAC4/5/6/7/9/10), class III (sirtuins), and class IV (HDAC11) [49]. Their expression patterns vary across tissues, and their regulatory effects on gene expression depend on cellular context and interactions with specific proteins [49]. The study by Kacper Maciejewski and colleagues [19] encompassed all HDAC family members and their transcriptomic profiles across 22 distinct tumor types available from publicly available The Cancer Genome Atlas (TCGA) datasets [50]. Several online bioinformatics applications, including GEPIA2, TISIDB, GSCA, Enrichr, and GSEA supported the analyses. 

The findings indicate that class I HDACs are uniformly upregulated across all tested tumor types compared to adjacent normal samples. In contrast, class III and IV HDACs are generally upregulated, but not in all tumor types. HDAC upregulation was observed to positively correlate with various clinicopathological parameters, such as tumor stage, grade, and TNM classification, but in a cancer- and gene-dependent manner. Most importantly, the analyses revealed that the expression patterns of class I HDACs, especially HDAC2, are strongly associated with stemness-related features (including published signatures) and an immunosuppressive tumor microenvironment across diverse solid tumors. Interestingly, the transcriptomic profiles of class II HDACs, and HDAC7 in particular, showed opposite associations. Class II HDACs demonstrated a negative correlation with stemness and a positive association with immune-related terms, including increased immune cell infiltration and expression of chemokines and their receptors, collectively indicating enhanced tumor immunogenicity. The study suggests that a therapeutic strategy combining class I HDAC inhibitors with ICI immunotherapy may help attenuate cancer stemness, while enhancing antitumor immunity [19].

In summary, the papers gathered in this Special Issue advance our understanding of how epigenetic and immune mechanisms shape tumor behavior, immune evasion, and responses to immunotherapy, while highlighting the growing value of epigenetic biomarkers in precision oncology. These articles demonstrate that epigenetic mechanisms influence not only intrinsic cancer cell programs, such as stemness, but also the composition and function of the tumor microenvironment. Moreover, the studies highlight the clinical potential of biomarkers such as PD-L1, hTERT methylation, circRNAs, and HDAC expression profiles for patient stratification and therapeutic decision-making. Despite the remarkable success of immunotherapies, many questions remain unanswered, including the mechanisms underlying primary and acquired resistance, the dynamic interaction between epigenetic states and antitumor immunity, and the reliability of current predictive biomarkers across heterogeneous tumors. Future research should focus on integrating epigenetic and immune profiling, validating novel biomarkers in prospective clinical trials, and developing innovative therapeutic strategies, including epigenetic modulators, circRNA-based technologies, and rational combinations of immunotherapy with epigenetic drugs, to further improve personalized cancer treatment.

## References

[1] Hanahan D., Weinberg R.A. (2011). Hallmarks of Cancer: The Next Generation. Cell.

[2] Flavahan W.A., Gaskell E., Bernstein B.E. (2017). Epigenetic Plasticity and the Hallmarks of Cancer. Science.

[3] Machnik M., Oleksiewicz U. (2020). Dynamic Signatures of the Epigenome: Friend or Foe?. Cells.

[4] Laisné M., Lupien M., Vallot C. (2025). Epigenomic Heterogeneity as a Source of Tumour Evolution. Nat. Rev. Cancer.

[5] Oleksiewicz U., Machnik M. (2022). Causes, Effects, and Clinical Implications of Perturbed Patterns within the Cancer Epigenome. Semin. Cancer Biol..

[6] Shah M.A., Denton E.L., Arrowsmith C.H., Lupien M., Schapira M. (2014). A Global Assessment of Cancer Genomic Alterations in Epigenetic Mechanisms. Epigenet. Chromatin.

[7] Verma A., Lindroth A.M. (2025). The Emerging Intertwined Activities of Metabolism and Epigenetics Unveils Culprits and Prospects in Cancer. Exp. Mol. Med..

[8] Xiao M., Yang H., Xu W., Ma S., Lin H., Zhu H., Liu L., Liu Y., Yang C., Xu Y. (2012). Inhibition of α-KG-Dependent Histone and DNA Demethylases by Fumarate and Succinate That Are Accumulated in Mutations of FH and SDH Tumor Suppressors. Genes Dev..

[9] Baylin S.B., Jones P.A. (2016). Epigenetic Determinants of Cancer. Cold Spring Harb. Perspect. Biol..

[10] Kroemer G., Chan T.A., Eggermont A.M.M., Galluzzi L. (2024). Immunosurveillance in Clinical Cancer Management. CA Cancer J. Clin..

[11] Kallingal A., Olszewski M., Maciejewska N., Brankiewicz W., Baginski M. (2023). Cancer Immune Escape: The Role of Antigen Presentation Machinery. J. Cancer Res. Clin. Oncol..

[12] Lee J., Kim E.H. (2023). Mechanisms Underlying Response and Resistance to Immune Checkpoint Blockade in Cancer Immunotherapy. Front. Oncol..

[13] Ma C., Cheng J., Gu J., Wang Q. (2025). Epigenetic Drugs in Cancer Therapy: Mechanisms, Immune Modulation, and Therapeutic Applications. Mol. Biomed..

[14] Bejarano L., Jordāo M.J.C., Joyce J.A. (2021). Therapeutic Targeting of the Tumor Microenvironment. Cancer Discov..

[15] Germanà E., Pepe L., Pizzimenti C., Ballato M., Pierconti F., Tuccari G., Ieni A., Giuffrè G., Fadda G., Fiorentino V. (2024). Programmed Cell Death Ligand 1 (PD-L1) Immunohistochemical Expression in Advanced Urothelial Bladder Carcinoma: An Updated Review with Clinical and Pathological Implications. Int. J. Mol. Sci..

[16] Poniewierska-Baran A., Sobolak K., Niedźwiedzka-Rystwej P., Plewa P., Pawlik A. (2024). Immunotherapy Based on Immune Checkpoint Molecules and Immune Checkpoint Inhibitors in Gastric Cancer–Narrative Review. Int. J. Mol. Sci..

[17] Santourlidis S., Araúzo-Bravo M.J., Brodell R.T., Hassan M., Bendhack M.L. (2024). hTERT Epigenetics Provides New Perspectives for Diagnosis and Evidence-Based Guidance of Chemotherapy in Cancer. Int. J. Mol. Sci..

[18] Włodarczyk K., Kuryło W., Pawłowska-Łachut A., Skiba W., Suszczyk D., Pieniądz P., Majewska M., Boniewska-Bernacka E., Wertel I. (2024). circRNAs in Endometrial Cancer—A Promising Biomarker: State of the Art. Int. J. Mol. Sci..

[19] Maciejewski K., Giers M., Oleksiewicz U., Czerwinska P. (2024). The Epigenetic Modifiers HDAC2 and HDAC7 Inversely Associate with Cancer Stemness and Immunity in Solid Tumors. Int. J. Mol. Sci..

[20] Stakhovskyi O., Kobyliak N., Voylenko O., Stakhovskyi E., Ponomarchuk R., Sulaieva O. (2022). Immune Microenvironment of Muscular-Invasive Urothelial Carcinoma: The Link to Tumor Immune Cycle and Prognosis. Cells.

[21] Bajorin D.F., Witjes J.A., Gschwend J.E., Schenker M., Valderrama B.P., Tomita Y., Bamias A., Lebret T., Shariat S.F., Park S.H. (2021). Adjuvant Nivolumab versus Placebo in Muscle-Invasive Urothelial Carcinoma. N. Engl. J. Med..

[22] Balar A.V., Castellano D.E., Grivas P., Vaughn D.J., Powles T., Vuky J., Fradet Y., Lee J.-L., Fong L., Vogelzang N.J. (2023). Efficacy and Safety of Pembrolizumab in Metastatic Urothelial Carcinoma: Results from KEYNOTE-045 and KEYNOTE-052 after up to 5 Years of Follow-Up. Ann. Oncol..

[23] Erber R., Hartmann A. (2020). Understanding PD-L1 Testing in Breast Cancer: A Practical Approach. Breast Care.

[24] Apolo A.B., Ellerton J.A., Infante J.R., Agrawal M., Gordon M.S., Aljumaily R., Gourdin T., Dirix L., Lee K.-W., Taylor M.H. (2020). Avelumab as Second-Line Therapy for Metastatic, Platinum-Treated Urothelial Carcinoma in the Phase Ib JAVELIN Solid Tumor Study: 2-Year Updated Efficacy and Safety Analysis. J. Immunother. Cancer.

[25] Balar A.V., Castellano D., O’Donnell P.H., Grivas P., Vuky J., Powles T., Plimack E.R., Hahn N.M., de Wit R., Pang L. (2017). First-Line Pembrolizumab in Cisplatin-Ineligible Patients with Locally Advanced and Unresectable or Metastatic Urothelial Cancer (KEYNOTE-052): A Multicentre, Single-Arm, Phase 2 Study. Lancet Oncol..

[26] Powles T., Walker J., Williams J.A., Bellmunt J. (2020). The Evolving Role of PD-L1 Testing in Patients with Metastatic Urothelial Carcinoma. Cancer Treat. Rev..

[27] Buchbinder E.I., Desai A. (2016). CTLA-4 and PD-1 Pathways: Similarities, Differences, and Implications of Their Inhibition. Am. J. Clin. Oncol..

[28] Kythreotou A., Siddique A., Mauri F.A., Bower M., Pinato D.J. (2018). PD-L1. J. Clin. Pathol..

[29] Qin S., Xu L., Yi M., Yu S., Wu K., Luo S. (2019). Novel Immune Checkpoint Targets: Moving beyond PD-1 and CTLA-4. Mol. Cancer.

[30] Dyck L., Mills K.H.G. (2017). Immune Checkpoints and Their Inhibition in Cancer and Infectious Diseases. Eur. J. Immunol..

[31] Fei S., Lu Y., Chen J., Qi J., Wu W., Wang B., Han Y., Wang K., Han X., Zhou H. (2023). Efficacy of PD-1 Inhibitors in First-Line Treatment for Advanced Gastroesophageal Junction and Gastric Cancer by Subgroups: A Systematic Review and Meta-Analysis. Chemotherapy.

[32] Janjigian Y.Y., Kawazoe A., Yañez P., Li N., Lonardi S., Kolesnik O., Barajas O., Bai Y., Shen L., Tang Y. (2021). The KEYNOTE-811 Trial of Dual PD-1 and HER2 Blockade in HER2-Positive Gastric Cancer. Nature.

[33] Wang F., Zhang X., Tang L., Wu Q., Cai M., Li Y., Qu X., Qiu H., Zhang Y., Ying J. (2024). The Chinese Society of Clinical Oncology (CSCO): Clinical Guidelines for the Diagnosis and Treatment of Gastric Cancer, 2023. Cancer Commun..

[34] Bhattacharyya G.S., Doval D.C., Desai C.J., Chaturvedi H., Sharma S., Somashekhar S.P. (2020). Overview of Breast Cancer and Implications of Overtreatment of Early-Stage Breast Cancer: An Indian Perspective. JCO Glob. Oncol..

[35] Ellinger J., Müller S.C., Stadler T.C., Jung A., von Ruecker A., Bastian P.J. (2011). The Role of Cell-Free Circulating DNA in the Diagnosis and Prognosis of Prostate Cancer. Urol. Oncol. Semin. Orig. Investig..

[36] Barthel F.P., Wei W., Tang M., Martinez-Ledesma E., Hu X., Amin S.B., Akdemir K.C., Seth S., Song X., Wang Q. (2017). Systematic Analysis of Telomere Length and Somatic Alterations in 31 Cancer Types. Nat. Genet..

[37] Lee D.D., Leão R., Komosa M., Gallo M., Zhang C.H., Lipman T., Remke M., Heidari A., Nunes N.M., Apolónio J.D. (2019). DNA Hypermethylation within *TERT* Promoter Upregulates TERT Expression in Cancer. J. Clin. Investig..

[38] Rowland T.J., Bonham A.J., Cech T.R. (2020). Allele-Specific Proximal Promoter Hypomethylation of the Telomerase Reverse Transcriptase Gene (TERT) Associates with TERT Expression in Multiple Cancers. Mol. Oncol..

[39] Malhotra S., Freeberg M.A., Winans S.J., Taylor J., Beemon K.L. (2018). A Novel Long Non-Coding RNA in the hTERT Promoter Region Regulates hTERT Expression. Non-Coding RNA.

[40] Ott P., Araúzo-Bravo M.J., Hoffmann M.J., Poyet C., Bendhack M.L., Santourlidis S., Erichsen L. (2022). Differential DNA Methylation of THOR and hTAPAS in the Regulation of hTERT and the Diagnosis of Cancer. Cancers.

[41] Geng X., Jia Y., Zhang Y., Shi L., Li Q., Zang A., Wang H. (2020). Circular RNA: Biogenesis, Degradation, Functions and Potential Roles in Mediating Resistance to Anticarcinogens. Epigenomics.

[42] Wei J., Li M., Xue C., Chen S., Zheng L., Deng H., Tang F., Li G., Xiong W., Zeng Z. (2023). Understanding the Roles and Regulation Patterns of circRNA on Its Host Gene in Tumorigenesis and Tumor Progression. J. Exp. Clin. Cancer Res..

[43] Shen Q., He T., Yuan H. (2019). Hsa_circ_0002577 Promotes Endometrial Carcinoma Progression via Regulating miR-197/CTNND1 Axis and Activating Wnt/β-Catenin Pathway. Cell Cycle.

[44] Shi R., Zhao R., Shen Y., Wei S., Zhang T., Zhang J., Shu W., Cheng S., Teng H., Wang H. (2024). IGF2BP2-Modified Circular RNA circCHD7 Promotes Endometrial Cancer Progression via Stabilizing PDGFRB and Activating JAK/STAT Signaling Pathway. Cancer Gene Ther..

[45] Li Z., Huang C., Bao C., Chen L., Lin M., Wang X., Zhong G., Yu B., Hu W., Dai L. (2015). Exon-Intron Circular RNAs Regulate Transcription in the Nucleus. Nat. Struct. Mol. Biol..

[46] Liu Z., Wang T., She Y., Wu K., Gu S., Li L., Dong C., Chen C., Zhou Y. (2021). N6-Methyladenosine-Modified circIGF2BP3 Inhibits CD8+ T-Cell Responses to Facilitate Tumor Immune Evasion by Promoting the Deubiquitination of PD-L1 in Non-Small Cell Lung Cancer. Mol. Cancer.

[47] Yu L.-L., Xiao Q., Yu B., Lv Q.-L., Liu Z.-Q., Yin J.-Y. (2023). CircRNAs in Tumor Immunity and Immunotherapy: Perspectives from Innate and Adaptive Immunity. Cancer Lett..

[48] Milazzo G., Mercatelli D., Di Muzio G., Triboli L., De Rosa P., Perini G., Giorgi F.M. (2020). Histone Deacetylases (HDACs): Evolution, Specificity, Role in Transcriptional Complexes, and Pharmacological Actionability. Genes.

[49] Seto E., Yoshida M. (2014). Erasers of Histone Acetylation: The Histone Deacetylase Enzymes. Cold Spring Harb. Perspect. Biol..

[50] Tomczak K., Czerwińska P., Wiznerowicz M. (2015). ReviewThe Cancer Genome Atlas (TCGA): An Immeasurable Source of Knowledge. Contemp. Oncol..

